# Remarkable Case of Cow-Pox

**Published:** 1808-12

**Authors:** John Mitchell

**Affiliations:** Kington, Herefordshire


### *Remarkable Case of Cow-Pox.*

A LAD, 14 years of age, was inoculated with the vaccine disease in his left arm, which went through its course in a very decided and regular manner; on the ninth day he had a slight attack of fever, the axillary glands enlarged and painful, continuing about two days; the disease receded from that time, and on the twenty-first day the scab had fallen off. Seven days after this time, and twenty-eight from the day of inoculation, the lad had a smart attack of fever, more violent than that which occurred on the ninth day. Upon investigating the cause, it evidently arose from three vaccine vesicles, of the size of those of the ninth day by inoculation, formed upon the lower part of his loins. The absorbent glands in each groin were swelled and painful. JOHN MITCHELL.

*Kington, Herefordshire, Oct.* 3, 1808.

N. B. No matter could possibly have been applied from other vesicle to the skin upon his back.

